# A Feasibility Study of Quantifying Longitudinal Brain Changes in Herpes Simplex Virus (HSV) Encephalitis Using Magnetic Resonance Imaging (MRI) and Stereology

**DOI:** 10.1371/journal.pone.0170215

**Published:** 2017-01-26

**Authors:** Sylviane Defres, Simon S. Keller, Kumar Das, Rishma Vidyasagar, Laura M. Parkes, Girvan Burnside, Michael Griffiths, Michael Kopelman, Neil Roberts, Tom Solomon, On behalf of ENCEPH UK study group

**Affiliations:** 1 Clinical Infection, microbiology and immunology, Institute of Infection and Global Health, University of Liverpool, Liverpool, United Kingdom; 2 Tropical and Infectious diseases Unit, Liverpool and Broadgreen University Hospital Trust, Liverpool, United Kingdom; 3 NIHR HPRU in Emerging and Zoonotic Infections, University of Liverpool, Institute of infection and Global Health, Waterhouse Building, Liverpool, United Kingdom; 4 The Department of Molecular and Clinical Pharmacology, Institute of Translational Medicine, University of Liverpool, Liverpool, United Kingdom; 5 The Department of Neuroradiology, The Walton Centre NHS Foundation Trust, Liverpool, Liverpool, United Kingdom; 6 Florey Institute of Neuroscience and mental health, Heidelberg, Victoria, Australia; 7 Department of Anatomy and Neuroscience, University of Melbourne, Victoria, Australia; 8 Division of Neuroscience and Experimental Psychology, Faculty of Biology, Medicine and Health, University of Manchester, Manchester, United Kingdom; 9 The department of Biostatistics, Institute of translational medicine, University of Liverpool, Liverpool, United Kingdom; 10 Alder Hey Children’s NHS Foundation Trust, Liverpool, United Kingdom; 11 Institute of Psychiatry, Kings College London, London, United Kingdom; 12 Medical Physics and Imaging Science, University of Edinburgh, Edinburgh, United Kingdom; Cincinnati Children's Hospital Medical Center, UNITED STATES

## Abstract

**Objectives:**

To assess whether it is feasible to quantify acute change in temporal lobe volume and total oedema volumes in herpes simplex virus (HSV) encephalitis as a preliminary to a trial of corticosteroid therapy.

**Methods:**

The study analysed serially acquired magnetic resonance images (MRI), of patients with acute HSV encephalitis who had neuroimaging repeated within four weeks of the first scan. We performed volumetric measurements of the left and right temporal lobes and of cerebral oedema visible on T_2_ weighted Fluid Attenuated Inversion Recovery (FLAIR) images using stereology in conjunction with point counting.

**Results:**

Temporal lobe volumes increased on average by 1.6% (standard deviation (SD 11%) in five patients who had not received corticosteroid therapy and decreased in two patients who had received corticosteroids by 8.5%. FLAIR hyperintensity volumes increased by 9% in patients not receiving treatment with corticosteroids and decreased by 29% in the two patients that had received corticosteroids.

**Conclusions:**

This study has shown it is feasible to quantify acute change in temporal lobe and total oedema volumes in HSV encephalitis and suggests a potential resolution of swelling in response to corticosteroid therapy. These techniques could be used as part of a randomized control trial to investigate the efficacy of corticosteroids for treating HSV encephalitis in conjunction with assessing clinical outcomes and could be of potential value in helping to predict the clinical outcomes of patients with HSV encephalitis.

## Introduction

Herpes simplex virus (HSV) encephalitis is the most common infectious cause of sporadic encephalitis [1–4]. It leads to haemorrhage, necrosis and extensive oedema, characteristically of the medial temporal lobes, and also additionally extends to affect other limbic areas including insular, cingulate and inferolateral frontal cortices [5]. These changes are best visualized by use of Magnetic Resonance Imaging (MRI) [6]. Although a rare disease with incidence rates of 1 per 250–500,000 [7], the impact of HSV is disproportionately large with huge socioeconomic demands on patients, their carers and the health services [8–9].

HSV encephalitis is currently treated with the antiviral drug aciclovir. Treatment with aciclovir has been reported to improve mortality from 70% to approximately 10–20% [7, 10–11]. However, survivors are left with a variety of sequelae including personality and behavioural changes, seizures, dysphasia and neuropsychological sequelae including memory problems [12–13].

Aciclovir stops viral replication but there is growing evidence that ongoing acute brain inflammation and swelling are major contributing factors to the pathogenesis of HSV encephalitis and may be related to poor outcome [14]. Corticosteroids are used to treat other neurological infections, including meningitis due to pneumococcus or tuberculosis [15–16]. Adjuvant corticosteroid therapy with aciclovir, in HSV encephalitis, was associated with improved clinical outcome at three months in a retrospective cohort study [17–18] and a reduction in severity of MRI abnormalities MRI in a mouse model [19].

In this preliminary study we sought to quantitatively examine serial MRI scans of people who have had HSV encephalitis to investigate changes in acute brain swelling over time, and potential resolution of swelling in response to corticosteroid therapy.

## Methods

### Participants

We performed a retrospective review of adult cases of HSV encephalitis from the ENCEPH-UK retrospective cohort study. The ENCEPH-UK study is an NIHR funded programme grant for applied research to better understand encephalitis and try to improve outcomes. Details of the programme can be found at www.encephuk.org. The retrospective arm of this programme builds on the UK Health Protection Agency (now called Public Health England) Aetiology of Encephalitis in England study [1]. In this study patients with encephalitis were recruited over a two year period from 2005 to 2007. Potential cases of encephalitis were identified from 24 hospitals across three regions in England. Patients with acute or sub-acute alteration in consciousness, cognition, personality or behaviour changes for more than 24 hours along with any two of: fever, prodromal illness, new onset seizures, focal neurological signs, pleocytosis (cerebrospinal fluid (CSF) white cell count of > 4 cells/μl), neuroimaging compatible with encephalitis or electroencephalogram (EEG) compatible with encephalitis, or any clinical suspicion of encephalitis when the investigations above have not been completed, were eligible. By using the same inclusion criteria further patients have been recruited to the retrospective cohort from 17 of the 33 participating sites in the prospective cohort, whose HSV encephalitis occurred between 2005 and 2013. The study protocols were approved by participating sites and the National Research Ethics Service (now part of the Health Research Authority) East Midlands Nottingham 1 committee and written consent for entry into the study was obtained from patients or an accompanying relative. Standardized case record forms for epidemiological, clinical, demographic, laboratory and radiological data are being used to collect data. Those meeting the case definitions for HSV encephalitis were included in this study, namely meeting the suspected encephalitis criteria above along with evidence of inflammation of brain parenchyma, and no alternative diagnosis made, and HSV type 1 identified by polymerase chain reaction (PCR) in the CSF.

### MRI Acquisitions

In order to be included in this study, each patient required a coronally-acquired T_2_- weighted Fluid Attenuated Inversion Recovery (FLAIR) scan that covered the entire brain. All MRI scanners had a 1.5 T field strength but the manufacturer and exact acquisition parameters for each FLAIR image varied but were similar at each site.Details of the scan parameters are in Table 1 and all scans were suitable for quantitative image analyses using the same stereological procedure. All scans were acquired as part of the routine clinical care of patients. Patients eligible for this study had to have had at least two MRI scans performed within four weeks of the admission scan on the assumption that MRI changes later than this may represent permanent damage and therefore not amenable to corticosteroid therapy. Each image was viewed carefully in terms of quality and potential presence of artefact and a judgement was made as to whether temporal lobe boundaries could be clearly seen. This assessment was performed by two members of the team. If the boundaries were not clearly seen then the scan was not included in the analysis.

**Table 1:** Details of the scan parameters for each patient.

Subject	MRI manufacturer	Scan	TE (ms)	TI (ms)	TR (ms)	Num slices	Voxel dimensions (mm)		
							x	y	z
1	GE Signa Excite 1.5T	1	111	1743	6975	28	0.47	0.47	4.0
		2	111	1743	6975	28	0.47	0.47	4.0
2	GE Signa Excite 1.5T	1	111	1743	6975	28	0.47	0.47	4.0
		2	111	1743	6975	28	0.47	0.47	4.0
3	Philips Achieva 1.5T	1	140	2800	11000	26	0.45	0.45	5.0
		2	140	2800	11000	26	0.45	0.45	5.0
		3	140	2800	11000	26	0.45	0.45	5.0
4	GE Discovery MR450 1.5T	1	128	2300	12000	25	0.43	0.43	5.0
		2	140	2800	11000	26	0.45	0.45	5.0
5	GE Genesis Signa 1.5T	1	161	2473	9897	26	0.94	0.94	5.0
		2	161	2473	9897	26	0.94	0.94	5.0
6	GE Genesis Signa 1.5T	1	161	2473	9897	28	0.47	0.47	5.0
		2	161	2473	9897	28	0.94	0.94	5.0

Legend: TE = echo time. TI = inversion time. TR = repetitions time. Num = number.

### MRI Analysis

**Fig 1:**
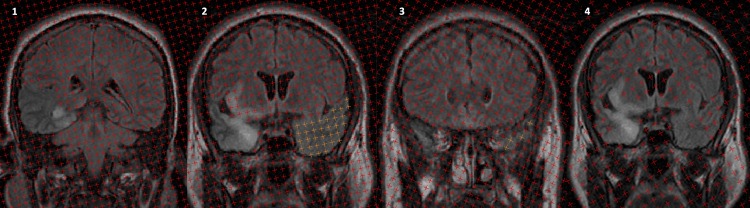
Image using the easymeasure stereology, point counting software showing the landmarks for temporal lobe measurements. (1) where the lateral ventricle splits into the temporal and frontal horns as the posterior limit for the right temporal lobe in a HSV encephalitis patient; (2) the mid point of the right temporal lobe measurement (in yellow the previously calculated left temporal lobe calculation); (3) the anterior limit of the temporal lobe and (4) the mid point section in (2) showing the volume of the overall damage including that outwith the temporal lobe, where HSV is seen to affect the right temporal lobe on this slice.

We performed volumetric measurements of the left and right temporal lobe, and of the volume of cerebral oedema visible on FLAIR images. Although both temporal lobes are frequently affected by HSV encephalitis often one is more affected than the other. FLAIR image hyperintensities were considered as likely oedema in this study. The volume estimates were obtained using the Cavalieri method of modern design-based stereology in conjunction with point counting.This is a mathematically unbiased and reproducible technique for volume estimation of brain compartments on MR images with high precision [20–22]. Stereological techniques require a rater to determine the number of test points that intersect a particular region-of-interest when an array of probes is overlaid onto the MR image. Stereological analysis was performed using Easymeasure software, as previously applied in other stereological studies of compartmental brain volumes [22–24]. Investigator one had recent training in stereology and performed the measurements without knowledge of time-point or treatment. Temporal lobe volume was estimated on the coronal FLAIR images using the same anatomical definitions as previously described [25–26]. The anterior limit of the temporal lobes was where the temporal pole disappeared prior to the orbits appearing, and the posterior limit was the coronal section where the lateral ventricle split into the temporal and frontal horns (approximately the same section on which the full profile of the crus of the fornix can be visualised) see Fig 1. To obtain a precise estimate of temporal lobe volume, stereological parameters (i.e., size of test probes, number of sections, etc.) were optimized to achieve a Coefficient of Error (CE) lower than 5%. CE is defined as the ratio of the square root of the variance of the volume estimator to its mean and provides information on the precision of each volume estimate and is dependent on the complexity and size of a structure. Detailed information on the estimation of the CE is provided elsewhere [22, 27]. Separation between test points on the square grid used for point counting was 7 pixels with slice intervals between 4.0 and 5.0mm (i.e. every MR section,). Temporal lobe transect area was obtained by multiplying the total number of points recorded by the area corresponding to each test point. An estimate of temporal lobe volume was obtained as the sum of the estimated areas of the structure transects on consecutive systematic sections multiplied by the distance between sections. Approximately 600 points were recorded on approximately 24 systematic random sections through the temporal lobe. All measurements were repeated by the first investigator in order to calculate intra-rater variability. For the purpose of calculating inter-rater variability, a second investigator, with 17 years-experience of imaging stereology, completed volume measurements of the temporal lobe in both hemispheres and the extent of oedema overall in all six cases without knowledge of time point or treatment.

Given that in HSVE brain damage is not always limited to the temporal lobes, the total volume of signal abnormality on T_2_-weighted FLAIR images, loosely defined as cerebral oedema, was also measured for each hemisphere of the brain using the same stereological approach and software described for the temporal lobe (see Fig 1 image d). Separation between test points on the square grid used for point counting was 10 pixels, and slice interval was between 4 and 5 mm (every MR section). The region of interest was based on the area of FLAIR image hyperintensity. All hyperintense FLAIR image voxels were classified as being oedema and as previously observed for the pattern of HSV encephalitis, were in the temporal and limbic regions. All images for FLAIR assessment were reviewed with a neuroradiologist blinded to clinical information.

Statistical analysis: All statistical analysis was carried out using the SAS statistical package (Version9.3). Intra- and inter-examiner reliability were assessed using intraclass correlation coefficients.

## Results

**Fig 2:**
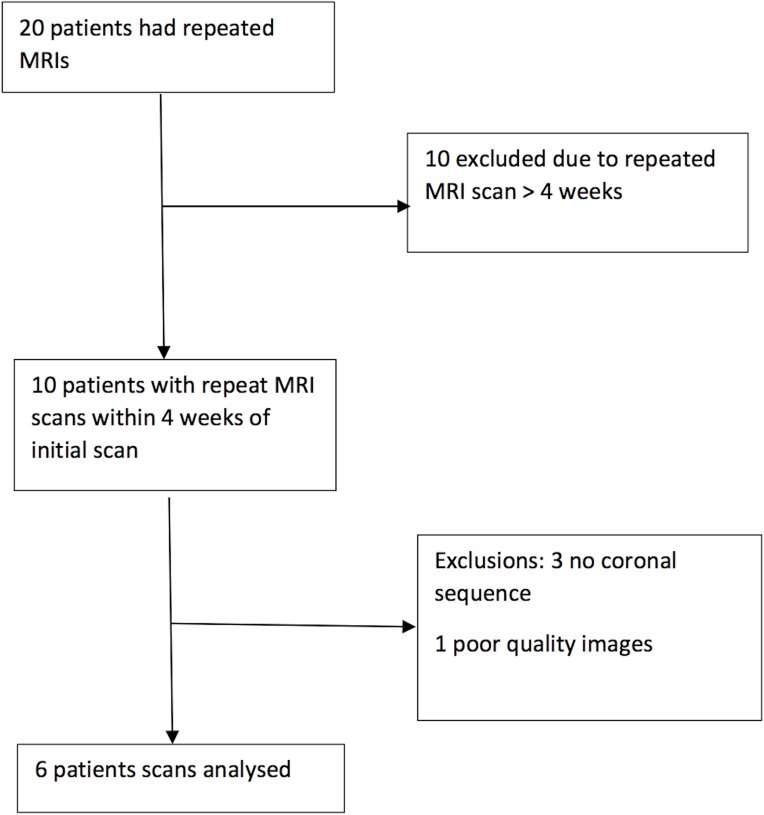
Flow chart of inclusion of patients in this study.

There were ten patients who had at least one repeat MRI within 4 weeks of the admission scan. Of these, four could not be analysed: three patients did not have coronal T2-weighted FLAIR scans performed on both sets of scans and one patient had poor quality images on the follow up T_2_-weighted FLAIR scan (Fig 2).

Patients were recruited initially on the basis of suspicion of encephalitis and subsequently confirmed as proven HSV-type 1 encephalitis on the grounds of evidence of inflammation on either cerebrospinal fluid (CSF) assessment, electroencephalogram (EEG) or Magnetic Resonance Imaging (MRI) findings. HSV type 1 was proven by polymerase chain reaction (PCR) in the CSF.

For the remaining six patients, two patients received corticosteroid therapy during their admission. One of these patients (Patient 3), had three MRI scans, thus enabling the 1^st^ period to be assessed as change in neuroimaging without corticosteroid therapy and the 2^nd^ interval as the change in neuroimaging post corticosteroid therapy. The demographics of the patients are described in Table 2.

**Table 2:** Characteristics of the patients on admission and their outcomes up to one year post discharge.

Patient	Age (yrs)	Sex	Symptom duration before admission (days)	Symptom triggering admission	GCS o/a	Delayed aciclovir (>48hrs)	Steroid therapy	ICU stay	LOS (days)	Discharge GOS	Discharge location	SF36 at 1 year
1	53	F	4	Seizure	9	Y	YES	Y	78	3	Rehab	Poor
2	19	F	28^a^	Seizure	15	N	NO	N	21	5	Home	Good
3	41	F	3	Focal neurology	14	N	YES	N	31	4	Home	Good
4	52	F	4	Headache	14	N	NO	Y	44	4	Home	Good
5	54	M	8	Coma	5	N	NO	N	34	5	Home	Fair
6	45	M	1	Seizure	3	Y	NO	Y	82	3	Rehab	Poor

Legend: yrs = years, M = male and F = female; GCS = Glasgow coma scale, a scale of consciousness from 3 to 15; o/a = on admission; ICU = intensive care unit; LOS = length of stay; GOS = Glasgow outcome score where 1 is dead and 5 is back to normal; SF36 = short form 36; a measure of general health status; Y = yes and N = no.

^a^ olfactory hallucinations occurred for 28 days prior to a seizure prompting admission

All patients experienced headache on admission but none had any other meningitic symptoms. All but one patient experienced fever on admission. The patient (Patient 2) that did not experience fever had been experiencing olfactory hallucinations prior to admission for many days, though a seizure triggered the admission to hospital. Olfactory hallucinations were also experienced by Patient 2. Focal neurology and expressive dysphasia was experienced in three and four patients respectively. None of the patients were immunocompromised on admission. Although lumbar punctures (LPs) were performed in all patients, two did not have an opening pressure recorded. For the others the initial opening pressure was either at the top end of normal or elevated and on subsequent LPs had reduced. CTs were available for all but one patient and reports described extensive swelling or swollen cortices suggestive of oedema. Patient 1 received a total of 17mg of intravenous dexamethsone over 6 days, commencing on day 6 of the admission and Patient 3 received a total of 316mg of intravenous dexamethasone over 19 days starting on day 4 of the admission.

Intra-rater variability, as measured by intraclass correlation coefficient (ICC), for temporal lobe and total oedema volume measurements was 0.998 and 0.995 respectively. The ICC for inter-rater variability for temporal lobe measurements was 0.976 and for total oedema measurements was 0.876.

Quantitative longitudinal analysis revealed that temporal lobe volume increased on average by 1.6% (standard deviation (SD 11%) in five patients who had not received corticosteroid therapy and decreased in two patients who had received corticosteroids by 8.5% (SD 0.5%).

Furthermore, quantitative analyses of changes in FLAIR hyperintensity volumes were found to increase on average by 9% (SD 15.1%) in patients not receiving treatment with corticosteroids and decreased on average by 29% (SD 1.5%) in the two patients that had received corticosteroids. These findings are tabulated in Table 3.

**Table 3:** A 2x2 table demonstrating whether volume changes increased or decreased in the two groups of patients, those treated with corticosteroids and those that were not.

Volume measurement	Treated group (2)	Not treated group (5)
Temporal lobes		
Increase	0	3
Decrease	2	2
Total FLAIR		
Increase	0	4
Decrease	2	1

**Fig 3:**
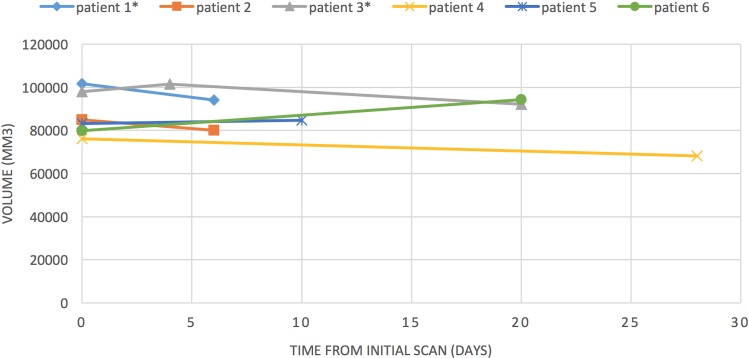
Change in volume of most affected temporal lobe over time. Patients 1 and 3 received corticosteroids during their care in hospital.

**Fig 4:**
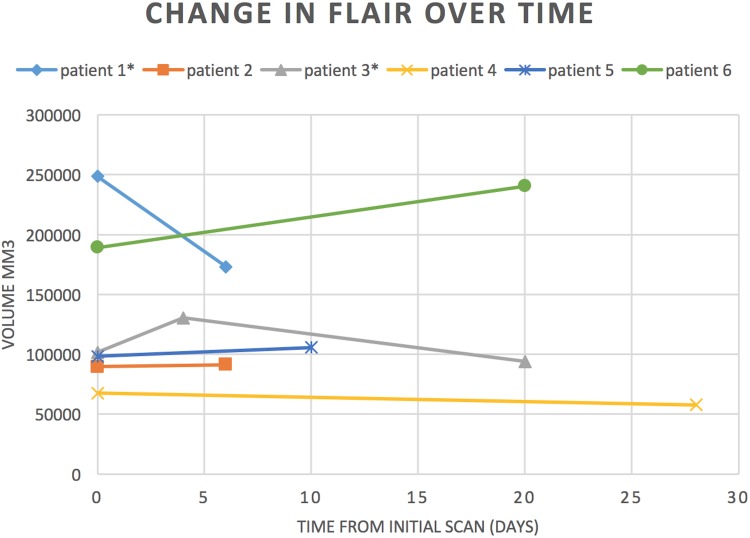
Change in volume of total oedema over time. Patients 1 and 3 received corticosteroids during their care in hospital.

**Fig 5:**
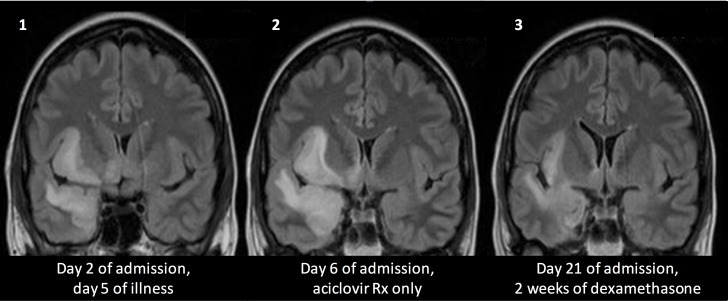
Coronal T2-weighted FLAIR images of a 45 year old woman with HSV encephalitis (Patient 3). On day 7 of admission a five-day course of corticosteroids was started. The same image slice is depicted from the day 2 scan (Image 1), from day 6 (Image 2) and from day 21 (Image 3)

The individual volume changes for each patient are presented in Table 4, Fig 3 and Fig 4. Fig 5. illustrates the changes for the patient who had three MRI scans showing the increase before corticosteroid therapy and the subsequent decrease after therapy.

**Table 4:** Magnetic resonance imaging abnormalities found and volume changes for each individual patients.

Patient	Time to 1^st^ scan from symptom onset (days)	Time to 1^st^ MRI from admission (days)	Time b/n MRI scans (days)	Abnormality on coronal T2w FLAIR				Steroid therapy
				Unilateral or Bilateral TL damage	Damage confined to TL	Proportionate volume change of worst affected TL	Volume change of oedematous region	
1	5	1	6	Bilateral	No	0.92	0.70	Yes
2	29	1	6	Unilateral	No	0.94	1.02	No
3a	6	3	4	Bilateral	No	1.04	1.28	No
3b	Na	NA	14^a^	Bilateral	No	0.91	0.73	Yes
4	6	2	27	Bilateral	No	0.90	0.85	No
5	14	6	11	Unilateral	No	1.02	1.08	No
6	2	1	34	Bilateral	No	1.18	1.27	No

Legend: TL = temporal lobe, MRI = magnetic resonance imaging, FLAIR = fluid attenuation inversion recovery; b/n = between. Patient 3a and 3b are the same person, 3a pre corticosteroid therapy and 3b post corticosteroid therapy, LOS length of stay. 3a is the period before corticosteroids, and 3b the period after corticosteroids were administered for patient 3.

^a^ this is the duration between the 2^nd^ and 3^rd^ scans.

The outcomes for the patients, in terms of length of stay, discharge Glasgow outcome score, where they were discharged to and their general health status at one year as measured by the SF36 are reported in Table 2.

## Discussion

In this small study of HSV encephalitis most patients on standard therapy had an increase in temporal lobe volume (three out of five) and of overall FLAIR-identified oedema (four out of five) during their acute hospital admission. However, a decrease in temporal lobe and oedema volume was observed in both patients who received corticosteroid therapy. Although the numbers are small in this study, the difference in the total oedema volumes between the patients not treated with corticosteroids and those that were, appears interesting and warrants further study

HSV encephalitis is a rare condition but its impact is considerable, including long hospital admissions, neurocognitive sequelae and prolonged periods off work. In one series only 14% of patients were reported as having complete recovery at six months by the Glasgow Outcome Score and in another study 45% of survivors who were in employment prior to their illness were unemployed at three years after their acute illness, [8–9]. Estimations of hospital stay costs in patients with encephalitis in the United States have shown a hospital admission in 2010 due to HSV encephalitis cost an average of $58,082 with total encephalitis associated hospitalisations amounting to approximately $2 billion [28]. In our series 33% were reported as having had a complete recovery at discharge and 50% having good outcomes at one year.

Previous neuroimaging studies in encephalitis have been mainly descriptive or correlate areas of abnormalities with clinical states or with aetiologies. In one study, initial abnormal CT scans, within three days of admission, was identified as a poor prognostic factor whilst another study identified ‘extensive’ MRI changes as being associated with poorer outcomes in HSV encephalitis [29–30]. Another study retrospectively examined MRI scans of patients with encephalitis and temporal lobe abnormalities suggesting that it is advantageous for predicting the aetiology to know whether abnormalities are located solely within one temporal lobe or are bilateral [31].

Verbal memory is particularly affected in patients that survive HSV encephalitis, which most likely reflects the fact that the damage is predominantly in the temporal lobes where new memories are formed [12–13, 32–34]. Bilateral temporal lobe swelling on MRI was associated with the severity of neuropsychological sequelae and in particular memory impairment [35]. In future larger studies, it will be important, therefore, to assess cognitive sequelae in conjunction with temporal lobe damage.

Few studies have quantified brain abnormalities in HSV encephalitis and fewer still have correlated these with clinical information. Some studies have performed correlations between different modalities of neuroimaging, for example between FLAIR images and Diffusion Weighted Imaging (DWI), and others have used voxel based morphometry to correlate with neurocognitive problems on research scans not typically acquired in routine clinical practice [36–37].

We found that our inter-rater correlation was better for temporal lobe measurements compared to total oedema volume measurements most likely because of the subjectivity associated with assessing areas of diffuse hyperintensity. Temporal lobe measurements have been standardized previously and HSV encephalitis is well known to cause damage in the temporal lobes. However, HSV commonly affects extra-temporal lobe regions and bihemispheric alterations are frequently observed. Measurement of temporal lobe volumes alone therefore would neglect significant areas of abnormality in HSV encephalitis. This was highlighted in the present study were larger changes in volumes were observed over time when assessing the whole area affected by HSV encephalitis and not solely the most affected temporal lobe. Indeed, in the two patients who received corticosteroids the decrease in temporal lobe volume was 8.5%. However, the decrease in volume of the area of oedema was 29% when the whole area of FLAIR hyperintensity was examined. From the data presented here there is a suggestion that corticosteroids can reduce damage caused by HSV encephalitis as viewed on MRI in the acute period of admission.

There are a number of limitations of this study. In particular it is retrospective, the numbers are small with limited ability to draw clinical correlations and the corticosteroids were not randomly administered. Indeed, the most clinically impaired patients may be those that get repeated neuroimaging or receive corticosteroids. Existing national and international guidelines do not suggest repeat neuroimaging in HSV encephalitis nor are corticosteroids recommended [38–39]. Therefore, the majority of patients with HSV encephalitis do not get either. Thus the use of three sets of MRI scans including before and after corticosteroid therapy was opportunistic in this feasibility study for both the treatment group and non-treatment group. Furthermore, MRIs were not standardised in this study as they were acquired in the context of a routine clinical setting. Also the surrogate marker of T_2_-weighted FLAIR imaging was used as an indication of oedema caused by HSV infection, but it is possible that the regions of hyperintesity were due to other underlying pathology such as haemorrhage. Furthermore, in the case of oedema, T_2_-weighted FLAIR alone cannot evaluate whether this is due to cytotoxic or vasogenic oedema. The use of additional MRI modalities, particularly DWI, would be important to further evaluate this. Although it is unorthodox to use the same participant for both a non-treatment group and a treatment group, this was a feasibility study for the stereology technique.

This was an opportunistic study of neuroimaging performed in routine clinical practice in HSV encephalitis. Furthermore each patient had their scans performed on the same scanner with the same scan parameters and therefore this should not affect serial intra-patient results.

Clinical outcomes should be evaluated in any future study both on the predictive value of neuroimaging on clinical manifestations in general, and in particular neuropsychological outcomes. In terms of the effectiveness of corticosteroids both on neuroimaging and also importantly on clinical outcomes, a randomized control trial would be necessary and to date none have been performed. Certainly the need for such a trial was identified in a systematic review in 2006 [40].

The randomised control trial, DexEnceph, which has opened to recruitment in January 2016, aims to answer the question of whether corticosteroids are beneficial and safe in treating HSV encephalitis. The trial will determine whether dexamethasone improves neuropsychological outcomes in sufferers of HSV encephalitis without allowing uncontrolled viral replication and whether corticosteroids will improve the neuroimaging, functional and quality of life outcomes. These developments are in turn likely to lead to obtaining a better understanding of the disease mechanisms in HSV encephalitis.

The findings from this study have shown that it is feasible to use routine clinical scans to evaluate and quantify the area of damage by HSV encephalitis and in the context of a larger randomized controlled trial could be valuable for the investigation of encephalitis and help predict clinical outcomes thus potentially enhancing the existing national guidelines on the management of HSV encephalitis.

### Ethical Standards

This study using data from people recruited to ENCEPH UK programme of studies was ethically approved by National Research Ethics Service (now part of Health Research Authority) East Midlands Nottingham 1 committee and has been performed in accordance with the ethical standards as laid down in the 1964 Declaration of Helsinki and its later amendments. All subjects gave their informed written consent prior to their inclusion in the study.

## References

[1] Granerod J, Ambrose HE, Davies NW, Clewley JP, Walsh AL, Morgan D et al Causes of encephalitis and differences in their clinical presentations in England: a multicentre, population-based prospective study. Lancet Infect Dis 2010;10:835–44 10.1016/S1473-3099(10)70222-X 20952256

[2] Hjalmarsson A, Blomqvist P, Skoldenberg B. Herpes simplex encephalitis in Sweden, 1990–2001: incidence, morbidity and mortality. Clin Infect Dis 2007;45:875–80. 10.1086/521262 17806053

[3] Mailles A, Stahl JP; Steering Committee and Investigators Group. Infectious encephalitis in France in 2007: a national prospective study. Clin Infect Dis 2009;49(12):1838–47. 10.1086/648419 19929384

[4] Bernard S, Mailles A, Stahl JP; Steering Committee and Investigators Group. Epidemiology of infectious encephalitis, differences between a prospective study and hospital discharge data. Epidemiol Infect 2013;141(11):2256–68. 10.1017/S0950268812002518 23168268 PMC9151439

[5] Corey L and Spear PG. Infections with Herpes Simplex Viruses. N Engl J Med 1986;314:749–757. 10.1056/NEJM198603203141205 3005859

[6] Schroth G, Gawehn J, Thron A, Vallbracht A, Voigt K. Early diagnosis of herpes simplex encephalitis by MRI. Neurology 1987;37(2):179–83. 3808297 10.1212/wnl.37.2.179

[7] Whitley RJ. Herpes simplex encephalitis: adolescents and adults. Antiviral research 2006;71:141–8 10.1016/j.antiviral.2006.04.002 16675036

[8] Mailles A, De Broucker T, Steering Committee and Investigators Group. Long-term outcome of patients presenting with acute infectious encephalitis of various causes in France. Clin Infect Dis 2012;54(10):1455–64. 10.1093/cid/cis226 22460967

[9] Raschilas F, Wolff M, Delatour F, Chaffaut C, De Broucker T, Chevret S et al Outcome of and prognostic factors for herpes simplex encephalitis in adult patients: results of a multicentre study. Clinical Infectious Diseases: an official publication of the Infectious diseases Society of America 2002;35:254–60. 10.1086/341405 12115090

[10] Sköldenberg B, Forsgren M, Alestig K, Bergstrom T, Burman L, Dahlqvist E, et al Acyclovir versus vidarabine in herpes simplex encephalitis. Randomised multicentre study in consecutive Swedish patients. Lancet 1984;2(8405):707–11. 6148470 10.1016/s0140-6736(84)92623-0

[11] Levitz RE. Herpes simplex encephalitis: a review. Heart Lung 1998;27(3):209–12. 9622408 10.1016/s0147-9563(98)90009-7

[12] Utley TF, Ogden JA, Gibb A, McGrath N, Anderson NE. The long-term neuropsychological outcomes of herpes simplex encephalitis in a series of unselected survivors. Neuropsychiatry Neuropsychol Behav Neurol 1997;10:180–9. 9297711

[13] Pewter SM, William WH, Haslam C, Kay JM. Neuropsychological and psychiatric profiles in acute encephalitis in adults. Neuropsychological rehabilitation 2007;17:478–505 10.1080/09602010701202238 17676531

[14] Skoldenberg B, Aurelius E, Hjalmarsson A, Sabri F, Forsgren M, Andersson B et al Incidence and pathogenesis of clinical relapse after herpes simplex encephalitis in adults. The journal of infectious diseases 2006;253:163–70. 10.1007/s00415-005-0941-6 16222428

[15] de Gans J, van de Beek D, European Dexamethasone in Adulthood Bacterial Meningitis Study Investigators. Dexamethasone in adults with bacterial meningitis. N Engl J Med 2002;347(20):1549–56. 12432041 10.1056/NEJMoa021334

[16] Thwaites GE, Nguyen DB, Nguyen HD, Hoang TQ, Do TT, Nguyen TC et al Dexamethasone for the treatment of tuberculous meningitis in adolescents and adults. N Engl J Med 2004;351:1741–51. 10.1056/NEJMoa040573 15496623

[17] Kamei S, Sekizawa T, Shiota H, Mizutani T, Itoyama Y, Takasu T et al Evaluation of combination therapy using acyclovir and corticosteroid in adult patients with herpes simplex virus encephalitis. J Neurol Neurosurg Psychiatry 2005;76:1544–1549 10.1136/jnnp.2004.049676 16227548 PMC1739396

[18] Tairi N, Kamei S, Morita A, Ishihara M, Miki K, Shiota H et al Predictors of a prolonged clinical course in adult patients with Herpes simplex Virus Encephalitis. Internal Medicine 2009;48:89–94. 19145052 10.2169/internalmedicine.48.1445

[19] Meyding-Lamade UK, Oberlinner C, Rau PR, Seyfer S, Heiland S, Sellner J et al Experimental herpes simplex virus encephalitis: A combination therapy of acyclovir and glucocorticoids reduces long term magnetic resonance imaging abnormalities. J Neurovirol 2003;9:118–125 10.1080/13550280390173373 12587075

[20] Doherty CP, Fitzsimons M, Holohan T, Mohamed HB, Farrell M, Meredith GE et al Accuracy and validity of stereology as a quantitative method for assessment of human temporal lobe volumes acquired by magnetic resonance imaging. Magn Reson Imaging 2000;18:1017–1025. 11121707 10.1016/s0730-725x(00)00185-5

[21] Garcia-Finana M, Keller SS, Roberts N. Confidence intervals for the volume of brain structures in Cavalieri sampling with local errors. J Neurosci Methods 2009;179:71–77. 10.1016/j.jneumeth.2009.01.026 19428514

[22] Keller SS, Baker G, Downes JJ, Roberts N. Quantitative MRI of the prefrontal cortex and executive function in patients with temporal lobe epilepsy. Epilepsy Beh 2009; 15: 186–195. 10.1016/j.yebeh.2009.03.005 19286475

[23] Keller SS, Highley JR, Garcia-Finana M, Slumming V, Rezaie R, Roberts N. Sulcal variability, stereological measurement and asymmetry of Broca's area on MR images. J Anat 2007;211, 534–555. 10.1111/j.1469-7580.2007.00793.x 17727624 PMC2375829

[24] Keller SS, Gerdes JS, Mohammadi S, Gerdes JS, Kugel H, Deppe K et al Volume estimation of the thalamus using freesurfer and stereology: consistency between methods. Neuroinformatics 2012;10:341–350. 10.1007/s12021-012-9147-0 22481382 PMC3464372

[25] Mackay CE, Roberts N, Mayes AR, Downes JJ, Foster JK, Mann D. An exploratory study of the relationship between face recognition memory and the volume of medial temporal lobe structures in healthy young males. Behav Neurol 1998;11: 3–20. 11568398 10.1155/1998/285061

[26] Mackay CE, Webb JA, Eldridge PR, Chadwick DW, Whitehouse GH, Roberts N. Quantitative magnetic resonance imaging in consecutive patients evaluated for surgical treatment of temporal lobe epilepsy. Magn Reson Imaging 2000;18:1187–1199. 11167039 10.1016/s0730-725x(00)00220-4

[27] Roberts N, Garden AS, Cruz-Orive LM, Whitehouse GH, Edwards RH. Estimation of fetal volume by magnetic resonance imaging and stereology. Br J Radiol 1994;67:1067–1077. 10.1259/0007-1285-67-803-1067 7820398

[28] Vora NM, Holman RC, Mehal JM, Steiner SA, Blanton J, Sejvar J. Burden of encephalitis-associated hospitalizations in the United States, 2014;1998–2010. Neurology 82(5):443–51. 24384647 10.1212/WNL.0000000000000086

[29] Riancho J, Delgado-Alvarado M, Sedano MJ, Polo MJ, Berciano J. Herpes simplex encephalitis: clinical presentation, neurological sequelae and new prognostic factors. Ten years of experience. Neurol Sci. 2013;34(10):1879–81. 10.1007/s10072-013-1475-9 23780666

[30] Sili U, Kaya A, Mert A, HSV Encephalitis Study Group. (2014) Herpes simplex virus encephalitis: clinical manifestations, diagnosis and outcome in 106 adult patients. J Clin Virol 60(2):112–8 10.1016/j.jcv.2014.03.010 24768322

[31] Chow FC, Glaser CA, Sheriff H, Xia D, Messenger S, Whitley R et al Use of clinical and neuroimaging characteristics to distinguish temporal lobe herpes simplex encephalitis from its mimics. Clin Infect Dis 2015;60(9):1377–83. 10.1093/cid/civ051 25637586 PMC4462661

[32] Hokkanen L, Launes J. Neuropsychological sequelae of acute onset sporadic viral encephalitis. Neuropsychological Rehabilitation 2000;17:450–77 10.1080/09602010601137039 17676530

[33] Colchester A, Kingsley D. Lasserson D, Kendall B, Bello F, Rush C et al Structural MRI volumetric analysis in patients with organic amnesia, 1: methods and comparative findings across diagnostic groups. J Neurol Neurosurg Psychiatry 2001;71:13–22 10.1136/jnnp.71.1.13 11413256 PMC1737485

[34] Kopelman MD, Lasserson D, Kingsley D, Bello F, Rush C, Stanhope N et al Structural MRI volumetric analysis in patients with organic amnesia, 2: correlations with anterograde memory and executive tests in 40 patients. J Neurol Neurosurg Psychiatry 2001;71:23–28 10.1136/jnnp.71.1.23 11413257 PMC1737465

[35] Reed LJ, Lasserson D, Marsden P, Bright P, Stanhope N, Kopelman MD. Correlations of regional cerebral metabolism with memory performance and executive function in patients with Herpes encephalitis or frontal lobe lesions. Neuropsychology 2005;19(5):555–565 10.1037/0894-4105.19.5.555 16187874

[36] Renard D, Nerrant E, Lechiche C. DWI and FLAIR imaging in herpes simplex encephalitis: a comparative and topographical analysis. J Neurol 2015;262(9):2101–5. 10.1007/s00415-015-7818-0 26092520

[37] Frisch S, Thiel F, Marschhauser A, Villringer A, Horstmann A, Schroeter ML. Identifying neural correlates of memory and language disturbances in herpes simplex encephalitis: a voxel-based morphometry (VBM) study. 2015;J Neurol 262(3):563–9. 10.1007/s00415-014-7604-4 25488475

[38] Tunkel AR, Glaser CA, Bloch KC, Sejvar JJ, Marra CM, Roos KL et al The management of encephalitis: clinical practice guidelines by the Infectious Diseases Society of America. Clin Infect Dis 2008;47:303–27. 10.1086/589747 18582201

[39] Solomon T, Michael BD, Smith PE, Sanderson F, Davies NW, National Encephalitis Guidelines Development and Stakeholder Groups. Management of suspected viral encephalitis in adults—Association of British Neurologists and British Infection Association National Guidelines. J Infect 2012;64(4):347–73 10.1016/j.jinf.2011.11.014 22120595

[40] Jacob A, Solomon T, Garner P. (2006) Corticosteroids in central nervous system infections In: Candelise L, Hughes R. Liberati A, Ultedhaag B, Warlow C (editors) Evidence-based neurology: Management of Neurological Disorders. Malden MA: Blackwell publishing, pp151-60.

